# A low power flexible dielectric barrier discharge disinfects surfaces and improves the action of hydrogen peroxide

**DOI:** 10.1038/s41598-021-84086-z

**Published:** 2021-02-25

**Authors:** Sophia Gershman, Maria B. Harreguy, Shurik Yatom, Yevgeny Raitses, Phillip Efthimion, Gal Haspel

**Affiliations:** 1 Princeton Plasma Physics Laboratory, Princeton, NJ USA; 2 Department of Biological Sciences, New Jersey Institute of Technology, Newark, NJ USA

**Keywords:** Plasma physics, Antimicrobials

## Abstract

There is an urgent need for disinfection and sterilization devices accessible to the public that can be fulfilled by innovative strategies for using cold atmospheric pressure plasmas. Here, we demonstrate a successful novel combination of a flexible printed circuit design of a dielectric barrier discharge (flex-DBD) with an environmentally safe chemical reagent for surface decontamination from bacterial contaminants. Flex-DBD operates in ambient air, atmospheric pressure, and room temperature without any additional gas flow at a power density not exceeding 0.5 W/cm^2^. The flex-DBD activation of a 3% hydrogen peroxide solution results in the reduction in the bacterial load of a surface contaminant of > 6log_10_ in 90 s, about 3log_10_ and 2log_10_ better than hydrogen peroxide alone or the flex-DBD alone, respectively, for the same treatment time. We propose that the synergy between plasma and hydrogen peroxide is based on the combined action of plasma-generated OH^**·**^ radicals in the hydrogen peroxide solution and the reactive nitrogen species supplied by the plasma effluent. A scavenger method verified a significant increase in OH^**·**^ concentration due to plasma treatment. Novel in-situ FTIR absorption spectra show the presence of O_3_, NO_2_, N_2_O, and other nitrogen species. Ozone dissolving in the H_2_O_2_ solution can effectively generate OH^**·**^ through a peroxone process. The addition of the reactive nitrogen species increases the disinfection efficiency of the hydroxyl radicals and other oxygen species. Hence, plasma activation of a low concentration hydrogen peroxide solution, using a hand-held flexible DBD device results in a dramatic improvement in disinfection.

## Introduction

There is an urgent need for wide use of sanitizing and disinfecting agents and techniques. Brought into focus by the current COVID-19 pandemic, it is no longer limited to medical, pharmaceutical, or food industry, but rather expanded to the decontamination of commonly used surfaces such as doorknobs and devices, such as masks, cell phones, and pens. Over the last two decades, cold atmospheric pressure plasmas (CAP) have seen rapid development in the areas of bacterial and viral inactivation and surface disinfection^1–8^. A recent review^8^ summarizes the achievements of a broad range of CAP plasma sources, including dielectric barrier discharges (DBD), that effectively inactivate bacteria, viruses, fungi, and bacterial spores. In spite of these achievements, the only sterilization method that involves plasma, which is currently recommended by the Centers for Disease Control and widely accepted in industry, is based on plasma activation of hydrogen peroxide vapor, one of the most effective and clean germicidal chemicals^9^. Hydrogen peroxide does not leave any dangerous residue because its decomposition products are water and oxygen. Here, we investigate the synergistic action of hydrogen peroxide and CAP implemented for the first time in a flexible device suitable for personal use and able to treat curved surfaces. We demonstrate faster disinfection than plasma or hydrogen peroxide alone in stable low power operation.

The disinfecting and even sterilization effectiveness of plasmas is due to their bio active properties such as reactive oxygen (ROS) and nitrogen species (RNS), electrons, currents, electric and electromagnetic fields, and UV rays^10–13^. The mechanisms of bacterial inactivation have been investigated by many groups but remain unclear. The chemical and electrical plasma properties may be affecting a bacterial cell in stages. The electrons and the electric field affect the cell membrane and aid in the cell penetration by the RNSs and some long-lived ROSs. ROS are involved in lipid peroxidation and other oxidative reactions damaging the cell membrane and aiding the transport of RNS/ROS into the cell. Inside the cell the ROS/RNS damage proteins, lipids, and the DNA. The combined effect of these processes is bacterial cell inactivation^8,14^.

Most of the work on medical and biological applications of DBDs has been conducted on one of three configurations, a floating electrode configuration, a plasma jet (floating electrode or two electrode), and a less common surface DBD^8,15–22^. In a floating electrode device, the high voltage electrode is encased in a dielectric material and the treated surface acts as a ground electrode^8,15,16^; the treated surface is exposed to high electric fields and fluxes of charged particles. The most extensively studied is the plasma jet, which uses power from pulsed dc to microwave range and where plasma effluent is carried by a gas flow to the treated surface. The plasma effluent is suitable for medical applications but requires a compressed gas supply^15,17–19^. Surface DBD has been primarily studied as an actuator for flow control in aeronautics applications and for large area surface modifications (ex.^20^). Introduction of devices based on flexible printed circuit design facilitated its applications in medical and biological fields^5,21–24^. The device studied here is also based on a flexible printed circuit design.

Atmospheric pressure plasmas have been shown to be effective for the decontamination of surfaces from bacteria and viruses, but the level and the rate of inactivation strongly depend on the biological species, experimental conditions, and the plasma source. For example, D-value (time for 1log10 reduction) is 225 s for the exposure to the gases produced by one DBD^25^, 150 s for another DBD^2^, 35 s for *E. coli* exposed to an atmospheric pressure helium/air glow discharge^3^, and 15 s for a paper-DBD^22^. The fast reduction D = 15 s was achieved by a single-use flexible DBD device using a printed patterned electrode on a paper substrate and operated at 2 kHz, 3.5 kV AC, 10 W. This is a disposable device^22^. This diversity of results and conditions makes it necessary for us to test the new device design presented here and quantify the disinfection of standard microorganisms.

Another variation on plasma disinfection is the use of low-pressure plasma activated hydrogen peroxide vapor^14,26,27^. Systems such as the low temperature sterilization systems by Sterlis Healthcare^9^ are widely accepted methods of sterilization of materials susceptible to high temperatures, humidity, and corrosion. In more recent studies, the addition of hydrogen peroxide has been explored to enhance plasma disinfection at atmospheric pressure^28–31^. Addition of H_2_O_2_ droplets into a corona discharge produced 6log_10_ reduction, and adding H_2_O_2_ vapor to the plasma effluent produced a reduction greater than 6log_10_ in the bacterial load and a significant reduction in biofilm and spores^28,30,31^. The dominant mechanisms responsible for the enhancement depend on the type of plasma, the state of H_2_O_2_. H_2_O_2_ vapor is ionized in a plasma to form H_2_O_2_^-^, while droplets may be negatively charged, a water solution of H_2_O_2_ is subject to the active species introduced by plasma into the solution akin to plasma activated water. Pure water is acidified by plasma enhancing the bactericidal effects, while buffered solutions such as phosphate buffer saline (PBS) maintain the pH level but are affected by the dissolved ozone, nitrates, and hydrogen peroxide radicals^32–34^.

The flex-DBD described here is safe to the touch, but unlike plasma jet devices, it does not need any additional gas supply or sophisticated power sources, and unlike a paper-DBD device it is capable of long-term stable operation. We demonstrate a fast disinfection effect of over 4log_10_ in less than 90 s on the standard bacteria, gram-negative *Escherichia coli* (*E. coli*). We also demonstrate the synergistic effect of the commonly available antiseptic, 3% H_2_O_2_, and the flex-DBD device, of surface decontamination > 6log_10_ in 90 s. The device tested here could be used to disinfect surfaces, personal items, and protection equipment such as masks.

## Methods

### Device assembly and electrical diagnostics

**Figure 1:**
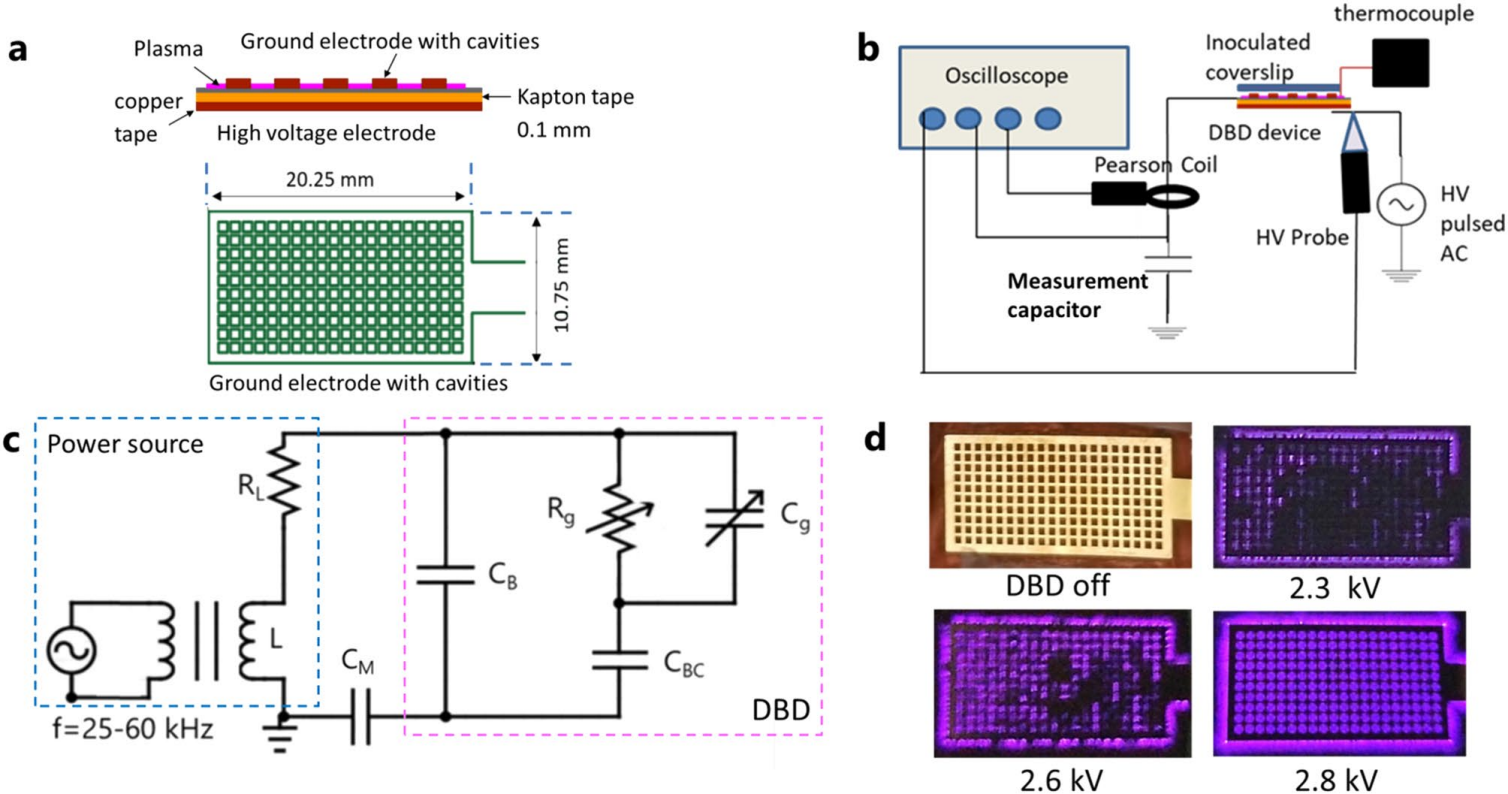
The flex-DBD is based on a printed circuit design and operates at < 3 kV, ~ 40 kHz, < 0.5 W/cm^2^ supplied by a portable power source. (**a**) The design of flex-DBD consists of conducting (copper) and insulating (polyimide) layers and a patterned ground electrode. (**b**) The experimental setup includes a portable pulsed ac high voltage (HV) source and monitoring apparatus (oscilloscope). The patterned electrode and HV source are globally grounded. A coverslip inoculated with a bacteria sample is placed on top of the ground electrode. (**c**) The equivalent circuit, where C_B_ is the capacitance of the flex-DBD device excluding the rectangular cavities, C_BC_ is the capacitance of the solid portion and C_g_ and R_g_ are the capacitance and resistance of the open air portion of the cavities, C_M_ is the measurement capacitor, and L and R_L_ are the inductance and the resistance of the secondary coil of the high voltage transformer of the high frequency power source. (**d**) As the applied voltage increases, a greater part of the surface of flex-DBD lights up.

The flex-DBD is based on a printed circuit design^5,22,24^. It consists of a layer of copper tape (0.127 mm thick, ] 16 mm × 26 mm) serving as a high voltage electrode and covered by a layer of Kapton polyimide tape (Kapton, DuPont), 100 µm thick, *∈*_*rel*_ ≈ 3.5, and a patterned ground electrode, copper (30 µm thick), electroless nickel immersion gold (ENIG) coated placed on top of the Kapton polyimide tape (Fig. 1a). The pattern on the ground electrode consists of 200 (10 × 20) square cavities, each 0.75 × 0.75 mm in size.

A regulated, 500 V–10 kV, 25–60 kHz, pulsed AC power source (PVM 500 AC, Information Unlimited) was used to generate the discharge (Fig. 1b). The patterned electrode of the flex-DBD was connected to the power-supply ground, and the copper foil electrode to the power-supply high voltage output transformer (Fig. 1b,c). We used a Tektronix D (2 GS/s, 250 MHz) oscilloscope to monitor the current, voltage, and charge transfer in the circuit during experiments: current to ground (Pearson Model 2877 Current Monitor, 1 V/A, 2 ns rise time); voltage at the high voltage (copper tape) electrode (Tektronix P6015 HV probe); and charge transferred was determined by measuring the voltage across a 10 nF capacitor (C_M_, Fig. 1b,c) connected in series on the ground side of the flex-DBD.

We operated the flex-DBD in resonance mode. The parallel connection between the DBD and the secondary coil on the high voltage output transformer has a resonance frequency, $\approx \frac{1}{{2\pi \sqrt {LC} }}$, where L, is the inductance of the secondary (Fig. 1c) and C is the capacitance of the flex-DBD. During operation the capacitance of the flex-DBD is approximately, C ≈ C_B_ + C_BC_. At the resonance frequency, the overall impedance of the L-C circuit as seen by the power source is at a maximum, therefore minimizing the current drawn and hence the power used by the device (Fig. 1c) and maximizing the voltage applied to the DBD, hence facilitating a discharge at lower power used by the power source. To start the device, we adjusted the frequency to a resonance value at a voltage amplitude below the starting voltage, then increased the applied voltage to the start the discharge (V_start_ = 1.9 kV amplitude), and readjusted the frequency to a new resonance value for the flex-DBD with the plasma on (Fig. 1d). The voltage was then increased until the entire surface of the flex-DBD appeared to glow to the naked eye (Fig. 1d, 2.8 kV). The resonance operating frequency was 42 ± 2 kHz. The variation in the resonance frequency is likely due to the slight differences in the hand-made devices and the operating conditions. We monitored the temperature of the grounded glowing face of the flex-DBD for several minutes prior to starting experiments to ensure that a steady-state condition was reached, and continued to monitor the temperature during experiments. Voltage amplitude and duty cycle were adjusted to maintain the temperature below 50 °C in steady-state operation. Except for the low power trial at 2 kV, the disinfection experiments were conducted with a voltage amplitude of 3 kV, a displacement current amplitude of 50 mA, a duty cycle of ~ 20%, and pulse repetition rate of 1 kHz. The voltage, current, and charge measurements were conducted during the sterilization experiments.

### Spectroscopy and imaging procedures

A PI-MAX 3 ICCD camera (Teledyne Princeton Instruments) was used for the fast imaging of the surface discharge (Supplementary material, Fig. S1a). The camera was triggered on a high current spike with minimal delay so that the shutter was open for the next current spike. We monitored the timing of the current spikes recorded by the Pearson coil and camera shutter on the oscilloscope and made the appropriate adjustments in the lengths of the connecting cables so that the images correlated to the current spikes.

Optical emission spectra (OES) of the light emitted by the flex-DBD were recorded in the range of 300–650 nm (Supplementary material, Fig. S1b) with an SpectraPro HRS-750 spectrometer coupled with PI-MAX 3 ICCD camera (both of Teledyne Princeton Instruments).

Fourier transform infrared absorption spectroscopy (FTIR AS) was used to assess the presence of more stable gaseous species in the plasma effluent from the flex-DBD. We placed the flex-DBD horizontally inside a test gas cell and turned it on (Supplementary material, Fig. S1c). The spectra were taken in the range of 700–3500 cm^−1^, at a resolution of 2 cm^−1^, using a FTIR-6800 with the MCT detector (Jasco). Each measurement of 64 scans took about 1 min. We then turned off the flex-DBD, flushed the instrument chamber and the gas cell with air, and recorded a new background spectrum prior to each measurement.

### Sterilization efficiency experiments

To demonstrate the disinfection ability of the flex-DBD, we tested the effectiveness of the device in reducing the bacterial load of *E. coli* 10-beta (New England Biolabs) and the standard *E. coli* AMS 198 (ATCC-11229). Bacteria were cultured following vendor’s instructions at 37 °C in Luria broth or Luria broth agar (both from Research Products International). For the surface test experiments, we used a suspension of the *E. coli* strain (OP-50-GFP, Caenorhabditis Genetics Center) that expresses cytoplasmic green fluorescent protein and forms a uniform bacterial lawn rather than discrete colonies.

**Figure 2:**
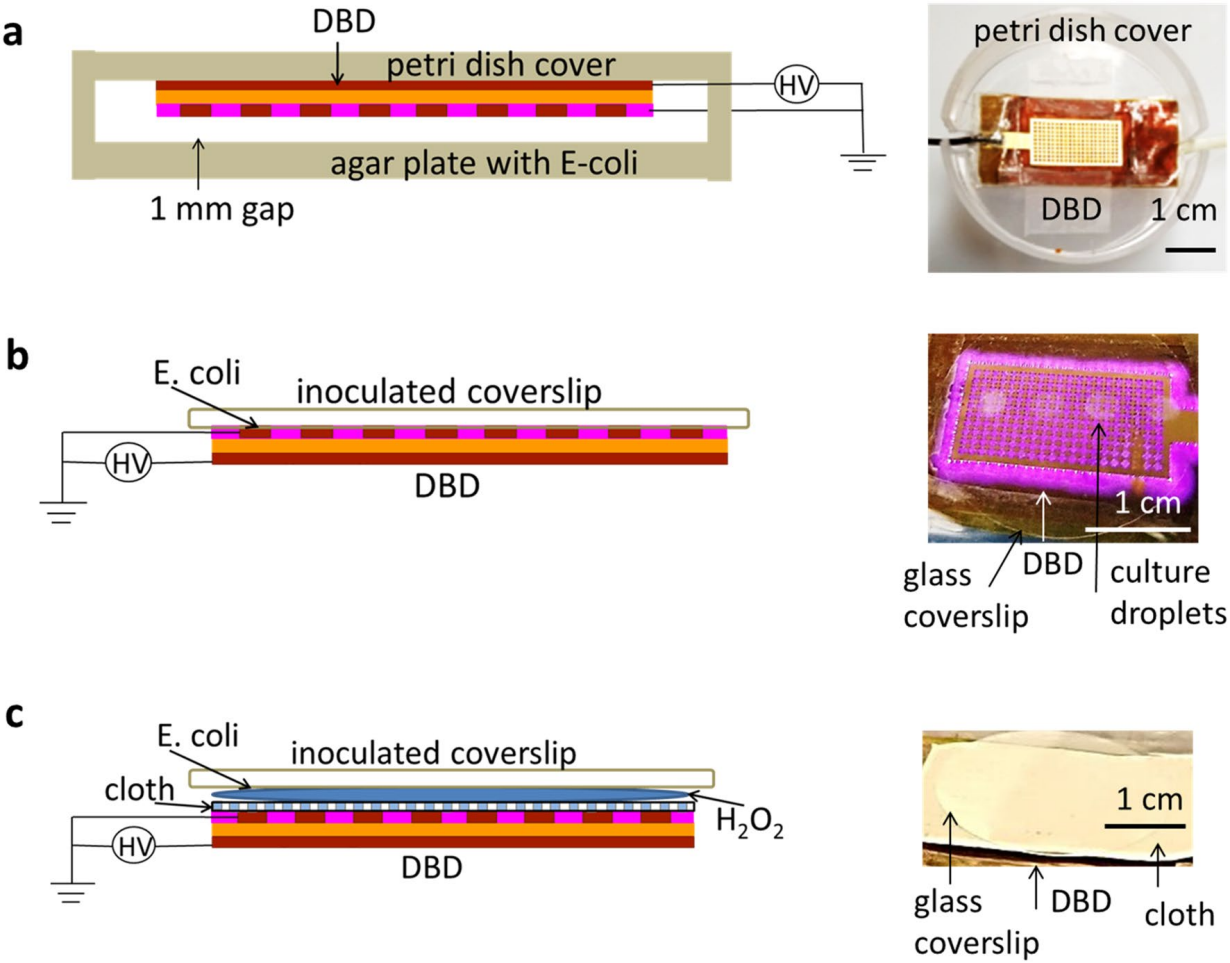
Flex-DBD at a short distance, in contact with a surface, or with a liquid disinfectant. (**a**) The flex-DBD is fixed to the inside of a petri dish lid. (**b**) Inoculated coverslip is directly in contact with the flex-DBD. (**c**) The flex-DBD is covered by a semi-permeable cloth with a liquid disinfectant on top; the inoculated cover slip is placed so that the bacterial culture is in contact with the disinfectant (20 µl dried culture, 10 µl 3% H_2_O_2_).

The flex-DBD was attached to a holder, or in other experiments, to the lid of a 60 mm petri dish (Fig. 2a). The experiments included the treatment of bacteria seeded in petri dishes, on a disposable textile type material, on metal (aluminum), and on glass (microscope cover slips). For treatment of bacterial plates, we spread 50 µl of a fresh bacteria culture on LB-agar plates and treated the plate surface with the flex-DBD attached to a lid, placed over the petri dish (Fig. 2a), for different amounts of time. We then incubated the plates overnight at 37 °C and visually compared them to untreated control and examined for areas that were clear of bacteria. To test the disinfection of the textile-type surfaces, 100 µl of *E. coli* OP-50 was spread on textile-like polyethylene material (Tyvek, DuPont). The petri dish cover with the flex-DBD attached was placed over the inoculated area. The petri dish cover used in this experiment was cut to maintain a 1–2 mm distance between the treated surface and the face of the flex-DBD. Each region was treated for a set amount of time and at the end of the treatment time, immediately stamped with an LB contact plate (Carolina Biological Supply Company). The untreated area was stamped as the control. The contact plates were incubated for 24 h at 37 °C. Qualitative results were assessed visually by observing GFP expression using a gel imaging station (FastGene Blue/Green LED GelPic Box, Nippon Genetics).

To quantify disinfection, bacteria (*E. coli* 10-beta, Standard *E. coli* AMS 198 or OP50-GFP) were inoculated on glass coverslips (25 mm diameter). Four droplets of 5 µl each (20 µl) of the bacterial culture (starting concentration 10^8^ CFU/ml) were placed onto each coverslip and allowed to dry for approximately 40 min. The slides with dry bacterial culture were then placed onto the flex-DBD with the inoculated side directly in contact with the discharge (Fig. 2b). The coverslips were treated for 10, 30, 90, and 270 s. At the end of the treatment time, the treated coverslip was placed in a centrifuge tube with 7.5 ml LB, enough to cover the coverslip. The tubes were vortexed on a medium setting for 20 s to recover the bacteria from the treated surface but not damage the cell membrane. The resulting bacterial suspension was spread on LB-agar plates and incubated for 24 h. Cultures were then counted and the number used to calculate the logarithmic reductions of bacterial concentration. All disinfection experiments were conducted with the flex-DBD operating at 3 kV, 20% duty cycle, and 40–50 °C.

We tested the efficiency of the flex-DBD disinfection in conjunction with a commonly available 3% solution of H_2_O_2_. The discharge in the flex-DBD is suppressed by water so we used a semipermeable polyethylene material (Tyvek, DuPont) to keep the H_2_O_2_ solution from the surface of the flex-DBD (Fig. 2c). Pieces of material were disinfected by soaking for 5 min in a 70% solution of isopropyl alcohol then dried thoroughly for at least 30 min. For each trial, we placed a piece of the sterile material on top of the operating flex-DBD and five 2 µl droplets of 3% H_2_O_2_ solution on top of the polyethylene material. We then placed an inoculated glass coverslip on top of the solution with the bacteria in contact with the solution (Fig. 2c). At the end of each treatment, we dropped both the coverslip and the cloth into a centrifuge tube with 7.5 ml of LB solution. The same recovery and plating procedure was used in all experiments. The controls for this experiment were inoculated but untreated coverslips, as well as the same procedure with H_2_O_2_ but with the flex-DBD remaining turned off. We also compared the disinfection efficiency of H_2_O_2_ aided only by the UV light produced by the flex-DBD. To block all the output from the plasma except light, we placed a thin film filter transparent down to 190 nm between the DBD and H_2_O_2_. Finally, to eliminate the operation temperature as a factor contributing to the disinfection process, we placed inoculated glass coverslips on a heating block at 47 °C and repeated the same disinfection procedures to determine the reduction in the bacterial load. We did not observe any reduction in the number of CFU/ml.

### Disinfection data analysis

To quantify bacterial load reduction, we imaged the treated plates after 24 h of growth and counted the number of bacterial colonies, interpreted as colony forming units (CFUs) in the plate-seeding solution. When possible, we used ImageJ (1.53c) software^35^ to count CFUs, otherwise we counted visually. The bacterial load in CFU/ml was calculated by multiplying the CFU count by 9375 (follows from 40 µl spread on each plate from coverslips washing volume of 7.5 ml LB broth, and original inoculation volume of 20 µl), and multiplying to account for any serial dilutions. The logarithmic reduction in the bacterial load was calculated as $log_{10} \left( {\frac{{N_{o} }}{N}} \right)$, where $N_{o}$ is the bacterial load at CFU/ml without any treatment (0 s coverslip), and N is the bacterial load at CFU/ml at each treatment time. All the experiments were performed in triplicates of samples and plates. Statistical significance between pairs of treatments was evaluated using repeated measures ANOVA.

### Chemical tests of the treated solutions

We determined the pH of the LB broth, Phosphate Buffer Saline solution (Sigma-Aldrich), and the 3% hydrogen peroxide solutions before and after the application of the flex-DBD, using the a pH meter (Aspera Instruments, model SX823-B, ± 0.01 pH) and pH test strips (Esee, ± 0.5) for amounts too small for the pH sensor.

We used two indicator strip tests to check the H_2_O_2_ production by the plasma, 2–200 ppm range test strips (Industrial Test Systems) to test the production of H_2_O_2_ in ≈20 µl of Luria Broth and a 1 – 10% range to check the changes in the concentration of in the 3% H_2_O_2_ solution used for the disinfection that combined the flex-DBD and H_2_O_2_.

We used a scavenger method to assess qualitatively the production of the OH^**·**^ in solutions during plasma treatment. We used coumarin (> 99%, Sigma-Aldrich) as a scavenger because it reacts with OH^**·**^ in solution to produce 7-hydroxycoumarin that fluoresces at 460 nm when excited at 390 nm^36^. Coumarin itself does not fluoresce in this spectral range. We prepared a 5 mM solution of coumarin in a 20 mM PBS by dissolving crystalline coumarin in the PBS solution at a pH9 and readjusting the pH of the resulting solution back to 7.4 with HCl^36^. We used three reference solutions: the coumarin stock solution, a solution prepared by adding equal amounts of coumarin solution and PBS, and a solution prepared by adding equal amounts of the coumarin stock solution and 3% hydrogen peroxide. The fluorescence of each solution was recorded at room temperature using an Ocean Optics Fluorescence/Absorption spectrometer. Coumarin/PBS solution and coumarin/hydrogen peroxide solutions were treated with the DBD discharge for 5 min, and the fluorescence of was recorded immediately following the treatment.

## Results and discussion

### Surface dielectric barrier discharge in a flex-DBD device

**Figure 3:**
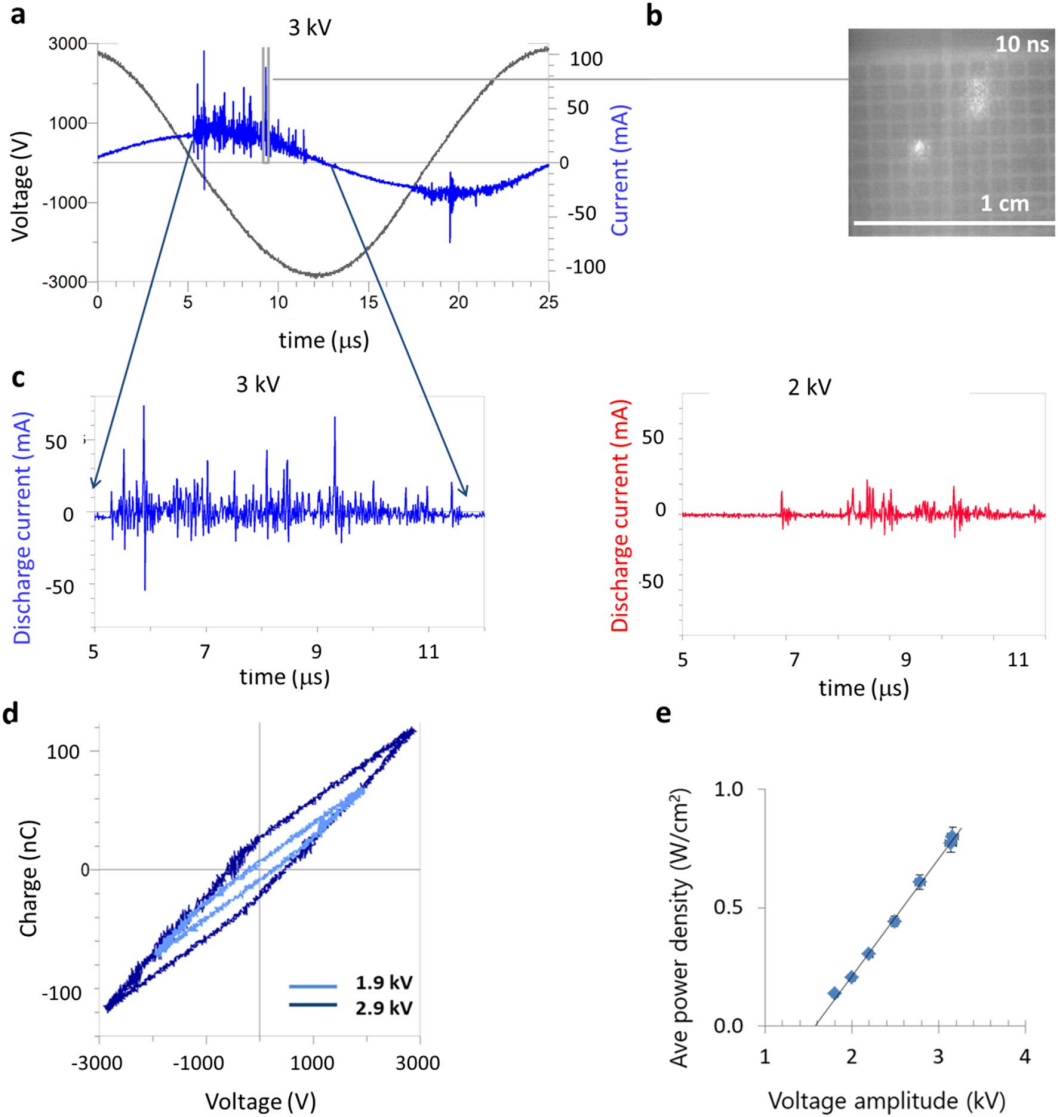
Electrical characteristics of the flex-DBD. (**a**) Current and voltage traces for one AC cycle. (**b**) Only three of ninety cavities are lit during a 10 ns exposure synchronized with an individual current pulse (marked in a). (**c**) The discharge current for one quarter of the cycle for applied voltage amplitudes of 3 kV and (**d**) for 2 kV. (**e**) From the Lissajous plots for one full cycle of the sinusoidal applied voltage we calculated that the 1.9 kV trial had 0.05 mJ/cycle and average discharge power of 0.3 W, while the 2.9 kV had 0.14 mJ/cycle and average discharge power of 0.75 W. (**f**) The average power increases linearly as a function of the applied voltage amplitude when the duty cycle and the frequency were kept constant at 20% and 40 kHz respectively.

A 40 kHz sinusoidal voltage, amplitude of 1.9–3 kV, was applied to the high voltage electrode for ~ 200 µs (20% duty cycle at 1 kHz repetition rate). A surface dielectric barrier discharge ignites inside the cavities and around the perimeter of the flex-DBD (Fig. 1d). The discharge propagates along the surface of the dielectric and eventually erodes the substrate of the ground electrode. The erosion pattern observed on post-run devices indicates that the discharge occurs in the center portion of each cavity leaving the angles intact. This is due to the electric field topology in the cavity with the maximum electric field E = V/r ~ 80 kV/cm, at the center of the circular cavity with a given radius (r). The erosion of the substrate of the patterned electrode happens over months of operation, possibly because only a few cells are lit at any time (apparent only at high temporal resolution imaging synchronized with the current spikes, Fig. 3b). During the discharge, the maximum current can be up to 100 mA for several tens of ns.

Water can form a conductive film that prevents charge accumulation on the electrode, preventing breakdown conditions^32,37–39^. This effect depends on the voltage rise time and breakdown voltage may be reached at sub-nanosecond rise times. However, in the flex-DBD, the voltage rise time is ≈7 µs, too slow to prevent the charge leakage from the conductors. The parts of the device that become moist will not light until dry. If that occurs when the power is on, heating due to the resistive and dielectric losses will self-dry the device and it will restart once the moisture evaporates. We used a water resistant material in the experiments with H_2_O_2_ solutions, to prevent the flex-DBD becoming wet while allowing active species from the plasma to reach the treated surface. This sensitivity to water is important for bio-related applications.

A typical current trace is comprised of a displacement current sinusoidal component of (42 ± 2) kHz and the superimposed sharp spikes 10 – 50 ns in duration corresponding to the discharges (Fig. 3a,c). The displacement current was subtracted from the total measured current to obtain the discharge current (Fig. 3c). The number of discharges, their overall duration, and their amplitude increase with increasing voltage (Fig. 3c). Although at 3 kV, the flex-DBD appeared completely lit, fast imaging triggered on a current spike demonstrates that during each current spike only a few bright regions are observed (Fig. 3b). Individual current spikes appear to correspond to isolated discharge events that appear randomly on the surface of the DBD. The number and the amplitude of the current spikes is not symmetrical in each half cycle of the AC current/voltage with a greater number of spikes occurring during the time when the mesh electrode acts as the anode, the voltage applied to the copper tape electrode is negative. In case of a positive mesh electrode, the electrons are able to flow into the anode and the current grows, but if the mesh electrode is negative, the electrons accumulate on the dielectric and the current stops resulting in lower current spikes. This asymmetry has been observed in plasma actuators that are a single edge surface DBD similar to the flex-DBD^20,35^. The number of individual discharges or current peaks varies depending on the maximum applied voltage (overvoltage). For example, the number of current peaks (over 10 mA) is 15 ± 8 at 2 kV and increases to 45 ± 8 for 3 kV (Fig. 3c). The greater number of current spikes results in a greater amount of charge transferred in the circuit as evident from the Lissajous plots (Fig. 3d).

The Lissajous plot has a two-slope shape with a slight asymmetry due to a greater number of more intense discharges for the negative voltage (positive patterned electrode). The energy dissipated in the circuit per one cycle can be calculated as the area of the Lissajous plot, and the power is then determined using the frequency, $f$, the duty cycle, $\nu ,$

1 $$P = f\nu \int QdV,$$

where Q is the charge measured by the capacitor probe and dV is the voltage obtained by the high voltage probe. For example, for the peak voltage of 1.9 kV the energy per cycle was 0.04 mJ/cycle. For the frequency of 41 kHz and a 20% duty cycle this gives the power of 0.3 W. For the max voltage of 2.9 kV the energy per cycle was 0.14 mJ/cycle, and the power, 1.1 W. The corresponding power density for the ~ 2 cm^2^ device is 0.15–0.5 W/cm^2^. The applied max AC voltage was varied from 1.6 kV to about 3 kV while keeping the frequency and the duty cycle constant. The resulting power varied linearly (Fig. 3e) with the applied voltage, which can be used as a calibration curve to set the desired power for a given device.

Increasing the operating voltage increases the discharge power and corresponds to an increase in the number of individual discharges and the production of plasma. Increasing the duty cycle increases the overall power consumption by the device, but does not change the number of individual discharges per cycle.

### Disinfection using the flex-DBD

**Figure 4:**
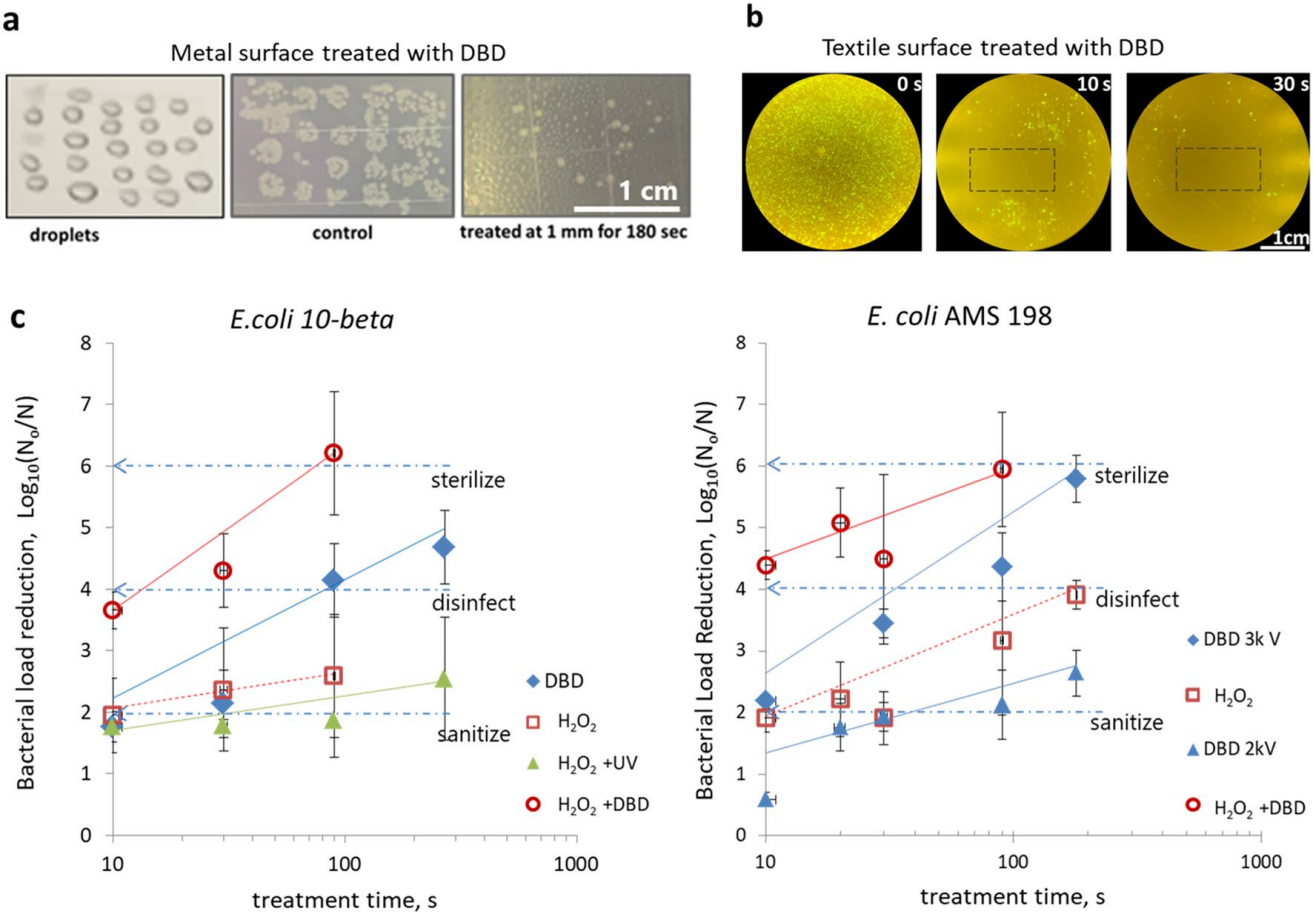
Flex-DBD device effectively reduces bacterial load and its effect is synergistic with hydrogen peroxide. (**a**) Aluminum surface inoculated with droplets of *E. coli* culture and stamped (“treated” photo). (**b**) Textile surface inoculated with *E. coli* (OP50-GFP) and stamped. (**c**) Reduction in the concentration of the *E. coli* colony forming units for two strains of *E. coli*. log_10_(N_o_/N), where N_o_ is the number of CFU/ml surviving in the untreated samples, N is the number of surviving CFU/ml that remain after the treatment for 10, 30, 90, and 270 s. Common scale bars in a and b are 1 cm. The error bars are one standard deviation.

To evaluate the effectiveness of the flex-DBD device in decontamination of surfaces from biological contaminants, we conducted qualitative and quantitative experiments. The qualitative experiments included the treatment of *E. coli* in petri dishes, the decontamination of inoculated aluminum and fabric surfaces (Fig. 4a,b); the quantitative bacterial load reduction was determined by treating bacterial culture dried onto glass coverslips (Fig. 4c).

We placed droplets of the bacterial culture on an aluminum surface, treated by exposure to the flex-DBD, and then stamped with contact plates. The flex-DBD effectively reduced the bacterial load when the DBD was placed 1 mm from the surface and operated at 3 kV and 44 kHz, duty cycle of 20%. The flex-DBD device was also effective at disinfecting textile-type textile-like polyethylene material. To assess the spatial extent of disinfection we uniformly inoculated the fabric with a transgenic *E. coli* strain that expresses green fluorescent protein (OP-50-GFP) and treated a 10 × 20 mm area with the same operating parameters remained. Only viable bacteria contain GFP and fluoresce when excited with blue light. Indeed, there was a marked reduction in GFP positive colonies around the treated area (Fig. 4b). The distance of the DBD from the surface is also important because reducing the distance from 1 mm above the surface to a direct contact with the ground electrode, increased the rate of inactivation of bacteria. We obtained a similar spatial pattern by treating *E. coli* bacterial culture dried onto the surface of a glass coverslip, following 30 s treatment with the flex-DBD.

To quantify the bactericidal effect of the flex-DBD we inoculated and dried glass coverslips and measured the surviving bacterial load in colony forming units per milliliter (CFU/ml). Treatment with the flex-DBD device reduced viable bacteria log_10_(N_o_/N) = 4.1 after 90 s (Fig. 4c). The flex-DBD was operated at a voltage of 3 kV and discharge power of 0.5 W/cm^2^, and the temperature of the grounded surface was below 50 °C. We repeated the inactivation of *E. coli* using the Standard *E. coli* strain AMC 198 (ATCC 11,229) (Fig. 4c). Two experiments were conducted, one using a lower voltage, 2 kV peak voltage and the temperature of the grounded surface T < 40 °C, and 3 kV peak voltage and T < 50 °C. The higher applied voltage resulted in faster (p = 0.003, ANOVA) inactivation of *E. coli*; log_10_(N_o_/N) = 5.8 after 180 s treatment (Fig. 4c) as compared to log_10_(N_o_/N) = 2.6 after 180 s, demonstrating a dependence on the flex-DBD peak voltage.

We calculated the 1log_10_ reduction (D value) for *E. coli* AMS 198 because the data is less variable than that of 10-beta, probably due to a greater control of the strain characteristics. At the start of the plasma treatment, the plasma affects the most susceptible bacteria that is located the closest to the plasma and hence is subjected to shorter-lived reactive plasma species. Hence the inactivation rate is the highest for the short treatment times. A linear fit to the treatment times of 10 s to 270 s give the times for 1log_10_ reduction, D = 74 ± 2 s with the correlation coefficient, R = 0.996.

We tested whether it has been the device operating temperature (40 °C and 50 °C) that caused disinfection of *E. coli* 10 Beta and *E. coli* AMC 198 strains. Instead of treatment with flex-DBD we incubated contaminated coverslips at 50 °C. We found no reduction in the bacterial load even at the longest exposure times of 180 s and 270 s. Therefore, the improvement in the disinfection at higher voltage may be attributed to plasma related effects such as the increase in the concentration of reactive species.

**Figure 5:**
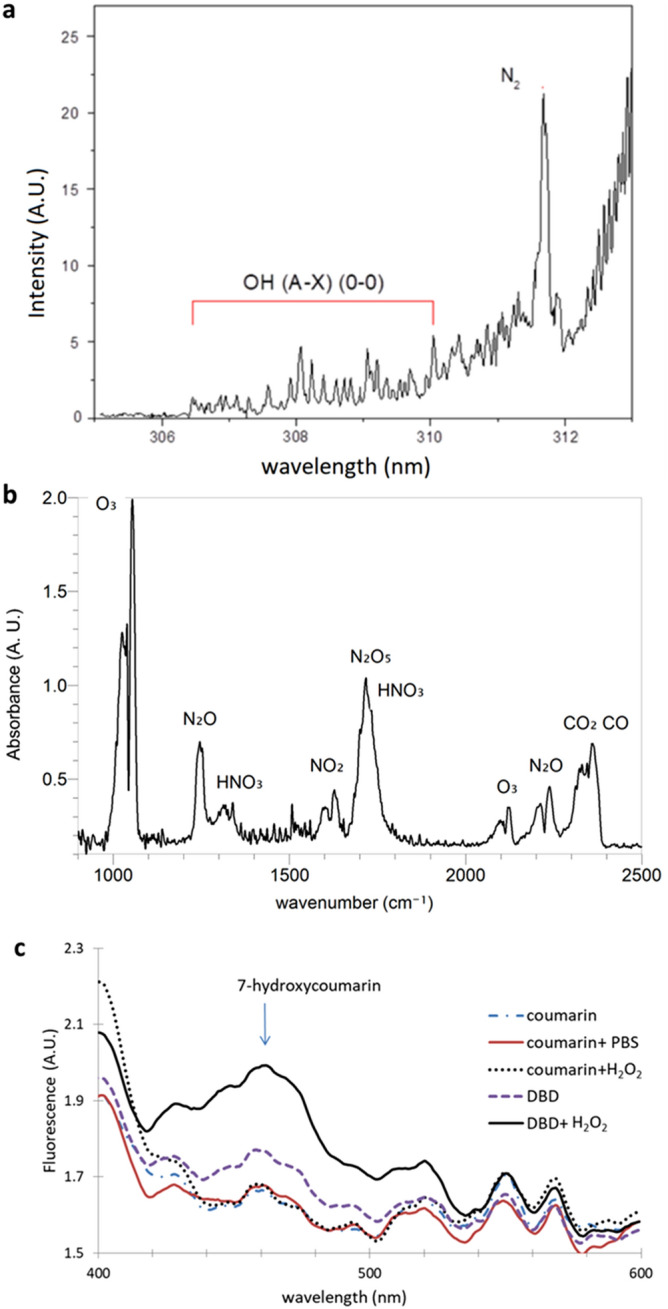
Spectral analysis of the flex-DBD: (**a**) Optical emission spectrum showing the OH^**·**^ emission band. (**b**) Infrared absorption spectrum showing the long-lived active species, O_3_, NO_2_, N_2_O, etc. produced in the discharge. Spectrum taken after 1 min of operation at the power and voltage used for treating bacteria. (**c**) Fluorescence of the 7-hydroxycoumarin indicating the presence of OH^·^ in solution. Solution of coumarin in PBS, and coumarin with H_2_O_2_ are not different from the coumarin in water that is used to produce the baseline spectra. Fluorescence intensity increased at 460 nm following plasma treatment (DBD) and further increased for plasma treated H_2_O_2_ solution (DBD + H_2_O_2_). Spectra were taken immediately after treating 1.5 ml liquid samples for 5 min at the same DBD setting. Each curve is an average of three trials.

Spectral analysis of the flex-DBD confirms the production of the OH^**·**^ (Fig. 5a) and a wide range of ROS and RNS (Fig. 5b) by the discharge. Hydroxyl radical is the highest oxidizer and it reacts with lipids in the cell membrane and oxidizes proteins and nucleic acids inside the cells. Because of its reactivity, it is very short-lived and needs to be produced at the site of action. Since the inoculated coverslips were in contact with the patterned side of the flex-DBD during treatment, we can speculate that the reactions of OH^**·**^ and other short-lived ROS are responsible for the fast initial rate of bacterial inactivation. Ozone and nitrogen oxides evident in the IR absorption spectrum can diffuse into the substrate and continue the disinfection process at a slower rate limited by the rate of diffusion and cellular processes^10–12^.

### Combined effect of plasma and hydrogen peroxide

To augment the disinfection we applied the flex-DBD treatment in combination with 3% H_2_O_2_ solution commonly available and, widely used for oral, skin, and wound disinfection. Applying the DBD together with H_2_O_2_ results in a 3.5 log_10_ reduction in the bacterial population in just 10 s and in > 5log_10_ reduction in 90 s (Fig. 4c). The combined disinfection effect of hydrogen peroxide and plasma is faster than either DBD alone (*p* = 0.04, repeated measures ANOVA test) or H_2_O_2_ alone (*p* = 0.03).

The antibacterial mechanism of H_2_O_2_ solutions alone is based on the production of the highly reactive hydroxyl radicals, but there is no appreciable equilibrium concentration of OH^**.**^ in the solution itself. We used a chemical scavenger method to detect the presence of the OH^.^ radical in 3% H_2_O_2_ solution and found that the concentration is of the same order as coumarin/PBS solutions that contain no hydrogen peroxide (Fig. 5c). The hydroxyl radical can be produced inside a cell by the Fenton reaction:

2 $${\text{Fe}}^{{{2} + }} + {\text{ H}}_{{2}} {\text{O}}_{{2}} \to {\text{Fe}}^{{{3} + }} + {\text{ OH}}^{ - } + {\text{ OH}}^{ \cdot }$$

The production of OH^**.**^ leads to oxidation and eventually to cell death. This disinfection by H_2_O_2_ alone depends on the concentration of the solution and can be concentration limited, slowing down as the hydrogen peroxide is used up^40^. Our results show that a bacterial load reduction due to the H_2_O_2_ alone (Fig. 4c) slows down faster than the corresponding DBD treatment (the times for 1log_10_ reduction, D = 139 ± 5 s, R = 0.97 for *E. coli* 10-beta; and D = 135 ± 5 s, R = 0.947 for *E. coli* AMC 198).

The sustained rate of reduction increases to D = 40 s (R = 0.86) when the treatment is carried out by both the DBD and the H_2_O_2_ solution (Fig. 4c) and reaches a > 6log10 reduction in just 90 s. In humid environments, air plasma produces hydroxyl radicals generally through the interactions of electrons and excited nitrogen with water vapor. But the hydroxyl radical reacts, oxidizes or recombines to form hydrogen peroxide on a microsecond scale^32,33,41^, which then diffuses into the solution, thus resulting in the production of H_2_O_2_ in the solution. The results of the indicator experiments support the increase of H_2_O_2_ concentration in both the Luria broth used for the *E. coli* suspensions and in the H_2_O_2_ solution used in the experiments with flex-DBD and H_2_O_2_. The application of flex-DBD to Luria broth for 90 s increases the concentration of H_2_O_2_ (and other oxidizing agents) to at least 100 ppm. Applying the flex-DBD directly to the H_2_O_2_ solution for 90 s treatment time, the concentration of increases the concentration of H_2_O_2_ from 3% before treatment to 5–10% after treatment.

Plasma can generate H_2_O_2_ in water solutions but the UV radiation from the plasma can also decompose the existing H_2_O_2_. To test whether the UV radiation from the flex-DBD was sufficient to explain the improved *E. coli* inactivation with H_2_O_2_ we blocked all plasma products except for the UV radiation with a UV filter. UV radiation improved the inactivation of bacteria in the first 30 s compared with H_2_O_2_ alone, but it did not achieve any additional reduction with increasing treatment time. After the first 30 s, the survival curve flattens (D > 250 s). This effect of UV radiation is insufficient to explain the improvement in the inactivation with H_2_O_2_ achieved by the addition of the DBD plasma. Hence the plasma, not UV radiation alone, improves the action of H_2_O_2_.

The remarkable synergy between plasma and H_2_O_2_ can be explained by the combination of the peroxone process with the RNS produced by the flex-DBD. The IR absorption spectrum is dominated by ozone and RNS. Peroxone is an advanced oxidation process that has been known for over 100 years. It involves the reactions of ozone and hydrogen peroxide that promote the production of OH^**·**^, which is a much more effective oxidizer than ozone alone^42^. Plasma-generated superoxide can also aid in the process of generating OH^**·**^ in solution. The scavenger method shows that plasma generates not only a stable hydrogen peroxide in a liquid solution but a measurable OH^**·**^ concentration (Fig. 5c). Plasma treatment of a H_2_O_2_ solution results in a significant increase in the concentration of OH^·^ in solution, well above plasma treatment alone. OH^**·**^ oxidation can lead to an easier penetration of the membrane by RNS that damage the proteins inside the cell and improve the overall disinfection process^10–12,43,44^. Hence the combination of plasma treatment with hydrogen peroxide is a powerful tool for disinfection of bacterial contaminants.

Although many years of investigation continue to demonstrate the potential of plasma in disinfection of biological and non-biological surfaces, the devices that have been accepted alongside the steam sterilization in medical and pharmaceutical industries are low pressure plasma-H_2_O_2_ vapor chambers^9,29,30^. Our results demonstrate that plasma disinfection could fill a technological gap not as a replacement for the standard bulk sterilization methods but in special niche applications and in personal consumer use^14^.

## Conclusions

Here we demonstrate the effectiveness of the first hand-held flex-DBD device that is suitable for personal use by untrained personnel. This flex-DBD device achieves fast disinfection, 4log_10_ reduction, in under 90 s and reaches > 5log_10_ in 270 s. Augmented with H_2_O_2_ flex-DBD it achieves high bacterial load reduction two times faster than alone: > 6log_10_ in 90 s with D = 2.5 s for the first 10 s and D = 30 s for 10 s – 180 s treatment times. These results are faster than UV and chemicals alone and faster than atmospheric pressure glow discharge, mesh DBD, and plasma activated water.

The synergy of plasma and hydrogen peroxide is due to plasma activation of H_2_O_2_. Our experimental results indicate that the mechanism of this activation is due to two factors. A peroxone process produces OH^**·**^ in H_2_O_2_ solution and the combined action of OH^·^ and RNS leads to enhanced disinfection. Novel in-situ FTIR AS measurements of the flex-DBD show the presence of ozone and RNS in the plasma effluent. The scavenger method demonstrates a significant increase in the OH^·^ concentration in the H_2_O_2_ solution. These measurements support the proposed mechanism.

The flex-DBD device can make effective disinfection accessible to the untrained public. It is safe in operation since the user facing components are grounded. The electrical measurements described above show that the flex-DBD operates consistently over prolonged periods, at least 15 min at a time for a total of more than 100 h of operation. The synergistic action with H_2_O_2_ reaches high levels of disinfection and opens exciting new possibilities for decontamination and treatment. This device can be used to disinfect personal protection equipment, hands, and frequently touched surfaces, as well as for wound treatment and other medical applications.

## Supplementary Information

Supplementary Information.

## Data Availability

The datasets generated during and/or analyzed during the current study are available from the corresponding author on reasonable request.
