# Supplementary material for: A low power flexible dielectric barrier discharge disinfects surfaces and improves the action of hydrogen peroxide
Source: Sci Rep. 2021 Feb 25;11:4626. doi: 10.1038/s41598-021-84086-z (PMC7907379; doi:10.1038/s41598-021-84086-z)
Supplement: Supplementary file 1 — Supplementary Information. [file 41598_2021_84086_MOESM1_ESM.pdf]

A low power flexible dielectric barrier discharge disinfects surfaces and improves the action of hydrogen peroxide.

Sophia Gershman[1*], Maria B. Harreguy[2], Shurik Yatom[1], Yevgeny Raitses[1], Phillip Efthimion[1], and Gal Haspel[2]
[1]Princeton Plasma Physics Laboratory, Princeton, NJ, USA
[2]Department of Biological Sciences, New Jersey Institute of Technology, Newark, NJ, USA
*sgershma@pppl.gov

Supplementary Information

Setups for spectroscopy and fast imaging

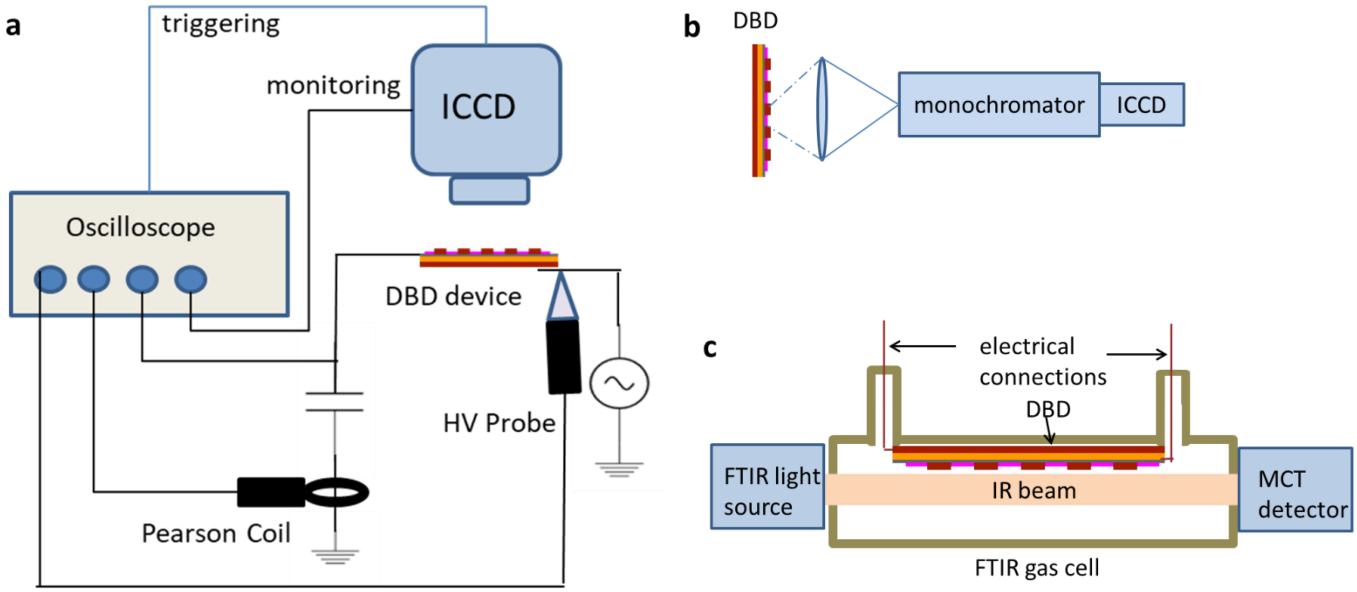

Figure S1 (**a**) Fast imaging. PIMTAX3 ICCD is triggered from the scope set to detect a current level about twice the AC amplitude which corresponds to a current spike. A minimum delay is used so that the image is taken during the next current spike. The gate width was 10 ns for all images. (**b**) We took the optical emission spectra face-on. (c) For the FTIR AS measurements, we positioned the flex-DBD inside a 10 cm gas cell in the sample chamber of the spectrometer. We used the MC detector and took 64 scans for each measurement at a resolution of 2 cm^{-1}.
